# P04.70. Determinants of botanical/specialty dietary supplement use among Hispanics participating in the 2007 National Health Interview Survey

**DOI:** 10.1186/1472-6882-12-S1-P340

**Published:** 2012-06-12

**Authors:** K Faurot, L Young, P Gardiner, D Zamora, S Gaylord

**Affiliations:** 1 UNC Epidemiology & Department of Physical Medicine & Rehabilitation, Chapel Hill, USA; 2 Department of Medicine, UNC School of Medicine, Chapel Hill, USA; 3 Department of Family Medicine, Boston Medical Center, Boston, USA; 4 UNC Program on Integrative Medicine, Department of Physical Medicine & Rehabilitation, Chapel Hill, USA

## Purpose

National surveys may underestimate prevalence of botanical/specialty dietary supplements (BDS) among Hispanics in the United States (US). We sought to examine prevalence and determinants of BDS use among Hispanics and variation by Latino background in the National Health Interview Survey (NHIS).

## Methods

We assessed the prevalence of BDS use in the past 12 months among respondents to the 2007 NHIS, a national probability sample of non-institutionalized US residents. Participants chose BDS from a list of products common in the general population. We examined prevalence by demographics and access to care (insurance) across racial and ethnic groups and Latino background groups: Mexican, Cuban, Puerto Rican/Dominican and Central/South American. We calculated prevalence odds ratios (POR) for BDS use with weighted logistic regression.

## Results

Controlling for age, sex, education, insurance status, years in the US, and US birthplace, Hispanics were less than half as likely to use BDS as non-Hispanic whites (9.8 vs. 21.3%; POR 0.37, 95% CI: 0.32, 0.43). Individuals reporting a Central/South American or mixed Latino background were more likely to use BDS than those reporting a Mexican background (POR 1.48 CI: 1.03, 2.14 and 1.87 CI: 1.41, 2.49, respectively). Among Hispanics, individuals 65-74 years old had more than three times the odds of BDS use as those 18-24 years old (POR 3.33 CI: 1.84, 6.05). Insurance status, education, US birthplace, and years in the US were not predictive of BDS use and had little effect on estimates by Latino background.

## Conclusion

Although BDS use appeared much less prevalent among Hispanics as compared with non-Hispanic whites, it likely represents a substantial underestimate: the NHIS BDS list excluded most herbal remedies used by Hispanics. National studies examining BDS common among Hispanics are needed to understand use patterns in this rapidly growing segment of the US population.

